# Undefined cellulase formulations hinder scientific reproducibility

**DOI:** 10.1186/s13068-017-0974-y

**Published:** 2017-11-28

**Authors:** Michael E. Himmel, Charles A. Abbas, John O. Baker, Edward A. Bayer, Yannick J. Bomble, Roman Brunecky, Xiaowen Chen, Claus Felby, Tina Jeoh, Rajeev Kumar, Barry V. McCleary, Brett I. Pletschke, Melvin P. Tucker, Charles E. Wyman, Stephen R. Decker

**Affiliations:** 1 Biosciences Center, National Renewable Energy Laboratory, Golden, CO 80401 USA; 2 Department of Food Science and Human Nutrition, University of Illinois, 905 S. Goodwin Ave., Urbana, IL 61801 USA; 3 Department of Biomolecular Sciences, The Weizmann Institute of Science, 76100 Rehovot, Israel; 4 National Bioenergy Center, National Renewable Energy Laboratory, Golden, CO 80401 USA; 5 Department of Geosciences and Natural Resource Management, University of Copenhagen, Rolighedsvej 23, 1958 Frederiksberg C, Denmark; 6 Department of Biological and Agricultural Engineering, University of California Davis, Davis, CA 95616 USA; 7 Bourns College of Engineering Center for Environmental Research and Technology, University of California, Riverside, 900 University Ave., Riverside, CA 92521 USA; 8 Megazyme, Irishtown, Bray, Co. Wicklow, A98 YV29 Ireland; 9 Department of Biochemistry and Microbiology, Rhodes University, PO Box 94, Grahamstown, 6140 South Africa

**Keywords:** Cellulase, Cellulose, Biofuels, Assays, Commercial cellulase formulations

## Abstract

In the shadow of a burgeoning biomass-to-fuels industry, biological conversion of lignocellulose to fermentable sugars in a cost-effective manner is key to the success of second-generation and advanced biofuel production. For the effective comparison of one cellulase preparation to another, cellulase assays are typically carried out with one or more engineered cellulase formulations or natural exoproteomes of known performance serving as positive controls. When these formulations have unknown composition, as is the case with several widely used commercial products, it becomes impossible to compare or reproduce work done today to work done in the future, where, for example, such preparations may not be available. Therefore, being a critical tenet of science publishing, experimental reproducibility is endangered by the continued use of these undisclosed products. We propose the introduction of standard procedures and materials to produce specific and reproducible cellulase formulations. These formulations are to serve as yardsticks to measure improvements and performance of new cellulase formulations.

## Background

Beginning with the discovery (or reaffirmation reports of plant extracts that degrade cellulose were reported in the late 1800s [1].) of biological cellulose degradation during World War II by the U.S. Army Natick Labs, the task of quantifying cellulase action has been challenging [2]. Most “cellulase” preparations are complex mixtures of several enzyme activities, some active on cellulose, some on other biomass components, such as xylan and pectin, and, especially in whole broths and commercial preparations, many component proteins are present without any apparent biomass-related activity. This complexity also makes it very difficult to obtain an accurate protein concentration value, as different components react variably to the various protein concentration reagents, and the types and ratios of the protein components vary significantly by the source microbe. True cellulases, i.e., the endocellulase, exocellulase, and β-d-glucosidase enzymes, work synergistically on insoluble substrates, so classical Michaelis–Menten enzyme kinetics do not apply. The substrate itself, cellulose from plant cell walls, displays compositional and structural variability based on source and processing methodology. Due to these and other complications, measurement of cellulase activity has always been challenging, in terms of both standardized assays and reproducibility.

Most early reports of cellulase kinetics relied on the simple measurement of soluble sugar released from filter paper [3–5]. In 1987, T.K. Ghose reported the development of the so-called “filter paper unit (FPU),” measured by a dilution series of the cellulase solution, wherein the dilution releasing 2.0 mg of equivalent glucose from a 50 mg coupon of Whatman No. 1 filter paper in 1 h was used to calculate FPU [6]. This measurement was established to provide a relatively simple assay to compare cellulases in different research laboratories, but is fraught with problems, such as low conversion extent (3.6%), complex reagents (i.e., 3,5-dinitrosalicylic acid), and operational issues, such as folded versus rolled filter paper and different tube diameters giving different results [7]. Notably, detection of FPUs below one FPU was not possible. The most problematic issue, however, is that it is a volumetric measure of activity typically used to assess highly concentrated commercial cellulases, requiring large dilutions which lead to large errors and giving little or no specific activity information.

For decades, experimental fungal and bacterial cellulases were prepared in the laboratories of the primary user. In the early 1990s, standard enzyme preparations appeared for sale from several enzyme producers, including Biocellulase TRI (Quest Intl.), Celluclast 1.5L (Novo Nordisk), Cellulase AP30K (Amano Enzyme), Cellulase TRL (Solvay Enzymes), Econase (Alko-EDC), Multifect CL, GC and Spezyme #1, #2, #3 (Genencor Intl.), and ultra-low microbial (ULM) (Iogen). These early commercial enzyme preparations were largely unadulterated microbial culture broths, usually filtered, concentrated, and stabilized for storage [8]. During this time, most reports of new cellulases or application of known enzymes to modified substrates included digestion data with one or more of these first-generation commercial cellulase preparations.

Today, advanced commercial cellulases, often formulated for increased efficacy on biomass, are used as reagents in many lignocellulose conversion experiments, including the evaluation of different biomass types for improved conversion properties, finding more effective biomass pretreatment methods, augmenting direct microbial conversion of biomass, and as a comparator or base formulation for experimentally improved cellulases. Most laboratories measuring cellulase performance generate hydrolysis progress curves on different substrates, where glucose release (cellobiases are often used to convert the cellobiose generated by cellobiohydrolases to glucose to simplify the quantitation of sugar release) is plotted against time. For industrial purposes, a “time-to-target” value is normally calculated. Just as critically, the enzymes used are more frequently loaded on a mass or protein, not FPU, basis. For example, the cellulase loading (in mg protein/g cellulose content) that gives a bioethanol process-relevant conversion target of cellulosic substrate (often 80%) has been determined for each enzyme formulation under study [9]. It should be noted that when using mg protein/g loadings, the method used to determine protein concentration should be specified, as there is variability between assays [10].

Commercial cellulases are often highly concentrated and formulated for protein solubility and stability in storage. Both precipitation of protein when diluted and loss of activity during long-term storage have been observed by our lab and others [11]. While the mechanisms behind these phenomena are not clear, these variables also impact activity comparisons. Furthermore, due to the variability of the biomass itself and the differential effects of various pretreatments on digestibility of biomass, some attempt should also be made to provide a reference digestion on an insoluble model substrate, such as commercially available microcrystalline cellulose (e.g., Avicel) or other appropriate model substrates. However, these model substrates have also been observed to have some composition and digestibility variations between batches. We note that the National Institute of Standards and Technology (NIST) has a series of biomass standards available and that these materials have shown stability in their composition over long-term storage; however, care must be taken to account for moisture content and substrate changes due to temperature fluctuations and storage conditions after being received [12].

## Observed problem

More recently, a new and disturbing trend has been observed in the reporting of cellulase action. To provide some context, it is a long-standing tenet in the publication of scientific data that ALL work described should be repeatable [13–15]. In other words, anyone reasonably skilled in the art should be able to repeat precisely the published results of another laboratory—regardless of the time delay. For cellulase research, however, the widespread use of newly developed, improved, and proprietary cellulase formulations possesses just such a publishing dilemma, as these have been developed for industrial use, not basic research. Certain commercial cellulase preparations, such as Novozymes’ Cellic^®^ series (Cellic^®^ Ctec2 and Ctec3) and Celluclast^®^ 1.5L, DuPont’s Accellerase^®^ line (Accellerase^®^ 1000, 1500, Duet, Trio), as well as cellulases from DSM, Primalco, Amano, and other enzyme suppliers that are used widely today for comparison or baselining purposes, have proprietary formulations. A quick PubMed search of the terms “Accellerase”, “Cellic”, and “Celluclast” yielded 45, 52, and 154 articles, respectively. Users are provided with these formulations only after signing agreements that state that they will not endeavor to reverse engineer or otherwise determine the enzyme complement constituting the formation. While this is certainly understandable and common from a commercial product protection point of view, it imposes significant limitations on the repeatability and understanding of biomass conversion research. As these formulations are not readily available to all researchers and their production is often discontinued in favor of new and improved variations, we propose that this practice violates the primary publishing tenet given above. This problem is further complicated by the limited shelf life of certain products, with activity losses occurring over time, even under proper storage conditions and exacerbated by large differences in measuring protein content using different methods and in different laboratories. In short, use of commercial cellulases as the only experimental means of comparison is nearly impossible to reproduce accurately and should be discouraged. But what are the alternatives?

## Conclusions and solutions

Despite all of its problems, the establishment of the filter paper assay as the standard cellulase test method was a marked improvement in comparability and consistency across cellulase research groups and eventually led to a more uniform and precise method based on sugar release over time by a known mass of enzymes. Similarly, we need a “standard cellulase assay” using a defined and reproducible cellulase formulation and consistent substrate, both of which are readily available to anyone in the field. Independent testing, characterization, and validation of commercial cellulases have always been, and will continue to be, critical to development of biomass conversion technology and a robust bioeconomy, but the lack of a consistent yardstick by which to measure improvements and performance of these crucial catalysts continues to confound and restrict advances in this field. It is time for a benchmark standard cellulase assay. This assay should use well-characterized substrate(s), preferably a “cellulose substrate,” to standardize enzyme preparations on a “defined” substrate, as well as a “biomass substrate” that could demonstrate performance of the enzymes under study on commercially relevant substrates. Avicel is often used as the cellulose substrate and could certainly become the cellulose standard material. A lignocellulosic biomass standard is more complicated, although it is likely that several variants would be needed, including a grass (corn stover or wheat straw), a hardwood (hybrid poplar), and perhaps a softwood (loblolly or southern white pine). Furthermore, the inclusion of cellulose-oxidizing LPMO enzymes in cellulase preparations may complicate a standard cellulase assay. The assay should make it possible to regulate the LPMO activity by controlling whether or not an electron donor for the LPMO is present. A finalized list is beyond the scope of this article but these suggestions are as good a place as any to start.

We propose that publishers refrain from accepting manuscripts wherein such proprietary formulations are used as the ONLY means for comparison. We also suggest that experimental solutions should be explored; for example, laboratories using cellulases routinely should (A) prepare these enzymes locally using designated type strains and standardized procedures for growing, processing, and quantifying fungal (*Trichoderma reesei* or others) and bacterial (*Clostridium thermocellum* or others) cellulases reproducibly and/or (B) establish an international consortium or single-source supplier for archiving, testing, characterizing, and distributing cellulase preparations of known performance and composition along with clearly defined protocols and standardized substrate(s). Option A presents its own problems of consistency, whereas alternative B would require significant administrative and production costs. Potential funding for this effort is not apparent, although potentially could be made available from a consortium of international governmental funding agencies, as part of a governmental standardizing agency (i.e., NIST), or from a trade association of cellulase producers. Even a single, standardized, and consistent commercial cellulase preparation would be desirable if it was constantly and readily available. The inclusion of one of these “standard” cellulase formulations should be a required control or internal standard when publishing cellulase assay results using commercial enzyme formulations. Such a standard will provide much more consistent comparisons between laboratories and cellulase formulations and expedite dissemination and implementation of improved cellulases, regardless of the source.
